# Chinese Medicine Formula Huashibaidu Granule Early Treatment for Mild COVID-19 Patients: An Unblinded, Cluster-Randomized Clinical Trial

**DOI:** 10.3389/fmed.2021.696976

**Published:** 2021-09-16

**Authors:** Chen Zhao, Li Li, Wei Yang, Wenliang Lv, Jian Wang, Jing Guo, Yu Dong, Nannan Shi, Cheng Lu, Zhiqiang Li, Zhan Shi, Renbo Chen, Ruili Huo, Qianzi Che, Yaxin Tian, Xinghua Xiang, Jian Wang, Junhui Zhou, Yongjun Bian, Suping Chen, Yang Chen, Yingying Chen, Xiaodong Cong, Guoju Dong, Lijie Hu, Jianxin Jiang, Luxing Leng, Bin Li, Dongxu Li, Hao Li, Jing Li, Wensheng Qi, Qing Miao, Huaxin Shi, Jiaheng Shi, Bing Wang, Gang Wang, Wei Wang, Yongyue Xian, Xiaolei Xie, Chunyan Xu, Ming Xu, Bei Yan, Jinliang Yang, Li Zhang, Zhenqi Zhou, Haoning Zhu, Yibai Xiong, Bin Liu, Luqi Huang

**Affiliations:** ^1^Institute of Basic Research in Clinical Medicine, China Academy of Chinese Medical Sciences, Beijing, China; ^2^Guanganmen Hospital, China Academy of Chinese Medical Sciences, Beijing, China; ^3^Department of Neurosurgery, Zhongnan Hospital of Wuhan University, Wuhan, China; ^4^China Academy of Chinese Medical Sciences, Beijing, China; ^5^Department of Traditional Chinese Medicine, Zhongnan Hospital of Wuhan University, Wuhan, China; ^6^Xiyuan Hospital, China Academy of Chinese Medical Sciences, Beijing, China

**Keywords:** COVID-19, Chinese medicine, early treatment, Fangcang Hospital, randomized clinical trial

## Abstract

**Background:** Previous research suggested that Chinese Medicine (CM) Formula Huashibaidu granule might shorten the disease course in coronavirus disease 2019 (COVID-19) patients. This research aimed to investigate the early treatment effect of Huashibaidu granule in well-managed patients with mild COVID-19.

**Methods:** An unblinded cluster-randomized clinical trial was conducted at the Dongxihu FangCang hospital. Two cabins were randomly allocated to a CM or control group, with 204 mild COVID-19 participants in each cabin. All participants received conventional treatment over a 7 day period, while the ones in CM group were additionally given Huashibaidu granule 10 g twice daily. Participants were followed up to their clinical endpoint. The primary outcome was worsening symptoms before the clinical endpoint. The secondary outcomes were cure and discharge before the clinical endpoint and alleviation of composite symptoms after the 7 days of treatment.

**Results:** All 408 participants were followed up to their clinical endpoint and included in statistical analysis. Baseline characteristics were comparable between the two groups (*P* > 0.05). The number of worsening patients in the CM group was 5 (2.5%), and that in the control group was 16 (7.8%) with a significant difference between groups (*P* = 0.014). Eight foreseeable mild adverse events occurred without statistical difference between groups (*P* = 0.151).

**Conclusion:** Seven days of early treatment with Huashibaidu granule reduced the likelihood of worsening symptoms in patients with mild COVID-19. Our study supports Huashibaidu granule as an active option for early treatment of mild COVID-19 in similar well-managed medical environments.

**Clinical Trial Registration:**www.chictr.org.cn/showproj.aspx?proj=49408, identifier: ChiCTR2000029763.

## Introduction

Coronavirus disease 2019 (COVID-19) was identified in December 2019 and spread to almost all regions of the world. According to the World Health Organization, more than 16 million cases have been confirmed and 640 thousand people died by July 27, 2020 (1). People of all races are susceptible to infection with COVID-19. The common clinical manifestations of early onset are fever and respiratory symptoms, and some patients will develop aggravated symptoms. In the absence of targeted medicine (2), patients with severe disease have poor prognosis and high mortality (3). Therefore, the development of active early treatment to limit disease progression in patients with mild COVID-19 has received considerable attention (4, 5).

Chinese medicine (CM) has a long history in treating infectious diseases with experience mainly recorded in ancient works such as *Treatise on Febrile Diseases (Shang-Han-Lun)* and *Differentiation of Febrile Diseases (Wen-Bing-Tiao-Bian)*. After many years of clinical verification, many CM formulae still guide treatment of acute infectious diseases today. In the early stage of an infectious disease outbreak without clear etiology, CM can diagnose and treat by analyzing the patient's symptoms and feelings, but there is generally a lack of evidence on the efficacy of this approach. Through clinical investigation, treatment in practice and expert consensus, the China National CM Rescue Team formed a CM formula known as Huashibaidu granule in late January, 2020 at Jinyintan Hospital, Wuhan city. Previous clinical observation showed that Huashibaidu granule could shorten disease course in patients (6), but no evidence yet supported the hypothesis that CM formula could improve the prognosis of patients with mild, early stage COVID-19 infection.

In February 2019, FangCang hospitals were set up in Wuhan city, China, as temporary hospitals converted from existing large-scale closed public buildings such as gymnasiums or exhibition centers (7). FangCang hospitals provided large numbers of hospital beds, so that patients with suspected symptoms could be quickly isolated after diagnosis with COVID-19, providing space for active treatment, vital provisions and social activity (8). The aim of the present study was to verify the effect of Huashibaidu granule in patients with mild, early stage well-managed COVID-19 in a FangCang hospital. It was hypothesized that treatment with Huashibaidu granule would result in better outcomes than with conventional treatment alone. For ethical reasons, all patients would receive standardized clinical conventional treatment.

## Materials and Methods

### Design Overview

From February 13 to March 7, 2020, a prospective, single centered, cluster-randomized, parallel controlled, unblinded clinical trial was conducted at Dongxihu FangCang hospital, Wuhan Keting, Hubei Province, China. Patients newly diagnosed with mild COVID-19 (*n* = 408) were enrolled from two isolation wards (referred to in this paper as “cabins”), and treated conventionally for 7 days with or without added Huashibaidu granule. The Ethics Review Committee of the Institute of Basic Research in Clinical Medicine, China Academy of Chinese Medical Sciences approved the clinical trial. All procedures performed in the study involving human participants were in accordance with the ethical standards of the institutional and/or national research committee and with the 1964 Helsinki declaration and its later amendments or comparable ethical standards. The study registration number on the Chinese Clinical Trial Registry is ChiCTR2000029763. This report is in accordance with the Consolidated Standards of Reporting Trials (CONSORT) statement (9).

### Participants

According to the fifth edition of the guideline for diagnosis and treatment of COVID-19 issued by the National Health Commission (NHC) of the People's Republic of China (10), COVID-19 patients were diagnosed with one of four different disease severity levels. Severe disease was defined as respiratory distress with respiratory rate ≥ 30 times / min, oxygen saturation ≤ 93% in resting state, or PaO2/FiO2 ≤ 300 mmHg. Very severe disease was defined as the occurrence of respiratory failure needing mechanical ventilation, shock, or other organ failure requiring intensive care unit (ICU) monitoring. Mild disease did not meet severe disease criteria and was categorized as ordinary (with lung imaging lesions) or gentle (without lung imaging lesions) disease level. Mild COVID-19 was diagnosed using a positive reverse-transcriptase–polymerase-chain-reaction (RT-PCR) assay for novel coronavirus via nasopharyngeal swab at the centralized isolation site, home or other location, and the patient was rapidly transferred to a FangCang hospital under isolation conditions. Patients with worsening symptoms would be transferred out of FangCang hospitals immediately and sent to other permanent hospitals for treatment.

In order to reduce infection risk from unnecessary contact between researchers and patients, information about the study was read by the researcher physicians in the FangCang hospital, then patients voluntarily and actively scanned a quick response (QR) code and completed a screening questionnaire, indicating that they consented to participate in the trial. Screened patients were given a unique electronic identification (ID) number. Patients aged 18–75 years with mild COVID-19 were enrolled. Patients with any of the following conditions were excluded: could not guarantee that medication would be taken during the trial observation period; more than 7 days after diagnosis; severe primary respiratory diseases; pregnant or planning pregnancy; malignant tumor or other malignant diseases; previous allergy to CM treatment; or current participant in other COVID-19 related trials.

All participants were observed up to their clinical endpoint. The clinical endpoints were defined as: cured and discharged from FangCang hospital; death in FangCang hospital; transferred out of the FangCang hospital with worsening COVID-19; or transferred due to the closure of FangCang hospital. Patients who voluntarily withdrew from the trial or transferred out for other reasons without cure were regarded as having dropped out of the trial.

### Randomization

Dongxihu FangCang hospital was divided into several physically isolated areas known as cabins. At the start of this trial, members of the research team were allocated to cabins B and C, where 742 mild COVID-19 patients had been admitted. Clinical investigation was immediately conducted to verify whether the patients in the two cabins were comparable. Based on the trial inclusion and exclusion criteria, researchers screened the patients, who completed informed consent as described above. Eligible patients were numbered according to ID number. R3.6.2 software was used for simple random sampling of patients in the two cabins by statistical researchers from China Center for Evidence-Based Traditional Chinese Medicine (CCEBTCM), then 204 patients were selected from each cabin, respectively. To maximize standardization of patient management and to minimize the burden of the trial on clinical practice, different interventions were implemented on cabin level, so one cabin was allocated to CM group and one to control by a simple randomized lottery. Both patients and researcher were unblinded.

### Intervention

Participants in both CM and control groups received conventional treatment in accordance with the NHC fifth edition guideline: bed rest, sufficient food and water intake, frequent monitoring of vital signs, and bedside oxygen therapy if necessary. As there was no evidence at the time of this trial that existing antiviral drugs were effective against COVID-19, Arbidol hydrochloride, as a compassionate therapy, could be given to patients under research physicians' discretion.

Patients in the CM group cabin were additionally given Huashibaidu granule during the 7-day treatment period. The daily dosage of the granule was converted from CM decoction pieces: Ma-huang (Herba Ephedrae) 6 g, Ku-xing-ren (Armeniacae Semen Amarum) 9 g, Shi-gao (Gypsum Fibrosum) 15 g, Gan-cao (Glycyrrhizae Radix et Rhizoma) 3 g, Huo-xiang (Pogostemonis Herba) 10 g, Hou-pu (Magnoliae Officinalis Cortex) 10 g, Cang-zhu (Atractylodis Rhizoma) 15 g, Cao-guo (Tsaoko Fructus) 10 g, Fa-ban-xia (Pinelliae Rhizoma Praeparatum) 9 g, Fu-ling (Poria) 15 g, Sheng-da-huang (Rhei Radix et Rhizoma) 5 g, Sheng-huang-qi (Astragali Radix) 10 g, Ting-li-zi (Descurainiae Semen Lepidii Semen) 10 g, Chi-shao (Paeoniae Radix Rubra) 10 g. The pharmacological action and main effective substances of each CM are shown in Supplementary Table 1; all CMs were tested for safety and quality. All Huashibaidu granule used within the trial was from the same batch and was donated by Huayi Pharmaceutical Co. Ltd. (Beijing, China) with Good Manufacturing Practice (GMP) qualification. The daily dose of CM was processed into 20 g granules packed equally in two opaque bags. One bag of granules was to be dissolved in 150–200 ml water at a temperature not lower than 80°C and taken about 20 min after breakfast and dinner. Patients were not given any other CM interventions during the 7-day treatment period, but they could continue to be given Huashibaidu granule or take other CM during the follow-up period if required. All intervention information is managed by the FangCang hospital information management electronic system.

### Outcomes and Data Management

The primary outcome was the rate of worsening before clinical endpoint occurrence. Worsening was defined as: patient diagnosed with severe or very severe disease type according to the NHC guideline (fifth edition), or death, or transferred for treatment following white blood cell or lymphocyte count significantly reduced on two or more consecutive routine blood tests. Secondary outcomes were discharge with cure and composite symptom outcome. Discharge outcome was determined from the hospital stay time and discharge rate of all patients before their clinical endpoint. The composite symptom outcome includes three symptoms indicated in the NHC guideline: fever, cough and fatigue. Disappearance of any of the three symptoms after the 7-day treatment period was defined as composite symptoms remission. Patients taking Abidor during the entire trial or CM after the 7-day treatment period were recorded.

Mild diarrhea is a known adverse event in previous clinical observation of Huashibaidu granule. Any other adverse events were recorded and handled in a timely fashion by research physicians inside the FangCang hospital. All adverse events were reported to the ethics committee within 24 h to determine whether the patient should continue to participate in the trial and whether they should be compensated.

Data on patients' worsening, discharge, transfer and drug usage were recorded in the FangCang hospital information management electronic system. Research physicians took photographs of the data using dedicated mobile phones, and transmitted them electronically to data managers at the CCEBTCM in Beijing. The data managers then entered the data onto the trial database and any uncertainties were fed back to the research physicians. The clinical endpoint determination committee in Beijing verified the authenticity of endpoints using photographs and electronic data. Each patient could scan the QR code using a personal mobile phone, by logging into the data management platform with their own IDs and inputting their symptom data themselves. The data quality control researchers in Beijing checked the electronic data against photographs and supervised the research physicians inside the FangCang hospital to remind the patients to report their symptoms data.

### Statistical Analysis

By analysis of data from 17,000 patients, the World Health Organization, found that worsening symptoms occur in about 18% of all COVID-19 patients (11). We anticipated that the rate could be reduced to 8% with CM. Using test efficiency of 1 - β = 0.8, and significance level of 0.05, an independent-groups sample size calculation indicated that 174 cases were needed in each group. We assumed that 15% of patients would be lost during the entire trial, so 408 participants would need to be enrolled.

Shapiro-Wilk normality test and homogeneity of variance test (F tests) were performed. Under the condition of normal distribution and homogeneity of variance, the continuous variables are expressed as mean (Standard Deviation, SD), and *t*-test was used for inter-group comparison; otherwise, they are expressed as medians (and interquartile range, IQR), and Wilcoxon rank sum test was used for inter-group comparison; categorical variables were expressed as number (%) and compared between group using the χ^2^ test or Fisher's exact test, as appropriate. Multivariate logistic regression was used to adjust for the primary outcome index. We then compared survival curves for time to discharge using Kaplan-Meier estimates and tested statistical significance using log-rank test. Missing symptom data on the 7th day were completed using the most recent data within 2 days. Independent sample tests were used for all statistical analyses with a significance level of *p* = 0.05. The statistical packages base, rcompanion and survival in R version-3.6.2 and SPSS 20.0 were used for statistical analysis and verification.

## Results

**Figure 1:**
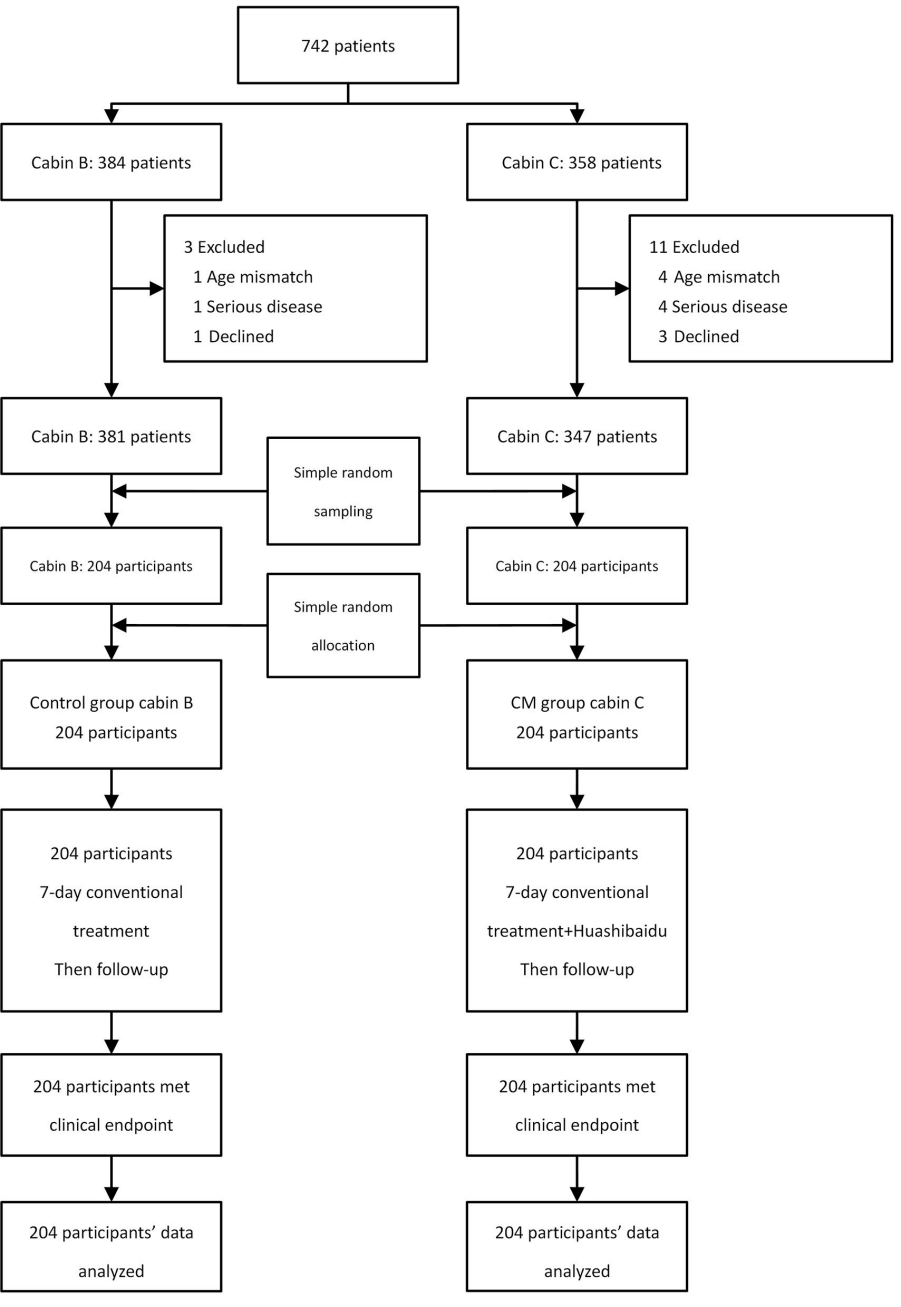
Study flow diagram.

After clinical investigation, 742 patients' demographic and source data were similar in the two cabins (*P* > 0.05), as shown in Table 1. Ten patients were excluded due to age and complications (three cases of cancer, one case with history of tuberculosis, and one case with asthma); four patients were excluded because they declined to scan the QR code to participate. Of the 728 patients who agreed to participate in the trial, 381 were in cabin B and 347 in cabin C. These were numbered and 204 patients were randomly sampled from each cabin. Through simple random allocation, 204 participants in cabin C were assigned to the CM group taking Huashibaidu granule for 7 days, and 204 participants in cabin B were the control group. By March 7, 2020, the Dongxihu FangCang hospital had completely closed, and all participants had met their clinical endpoint with no dropout, as shown in Figure 1. Baseline characteristics of the two groups were comparable (*P* > 0.05), as shown in Table 2.

**Table 1:** Demographic and source of patients in cabins B and C of Dongxihu FangCang Hospital before random sampling.

	**Cabin B (*n* = 384)**	**Cabin C (*n* = 358)**	***P-*value**
Age, median (IQR), y	50.0 (39.0, 59.3)	52.0 (39.3, 60.8)	0.284
Female sex, *n* (%)	190 (49.5%)	184 (51.4%)	0.602
Patients source^*^			0.507
Centralized isolation site, *n* (%)	41 (20.8%)	33 (17.7%)	
Others, *n* (%)	343 (89.3%)	325 (90.8%)	
Place of birth			0.076
Wuhan, *n* (%)	296 (77.1%)	299 (83.5%)	
Hubei Province excluding Wuhan, *n* (%)	65 (16.9%)	41 (11.5%)	
Outside Hubei Province, *n* (%)	23 (6.0%)	18 (5.0%)	

*IQR, interquartile range*.

* *Some patients were of unknown source, but were not from the centralized isolation site, so they were categorized as “others.” There were 187 such patients in CM group cabin C and 172 in control group cabin B*.

**Table 2:** Baseline characteristics of patients in the randomly allocated groups.

	**CM group cabin C (*n* = 204)**	**Control group cabin B (*n* = 204)**	***P-*value**
Age, median (IQR), y	52.0 (39.0, 58.0)	49 (37.8, 58.0)	0.134
Female sex, *n* (%)	116 (56.9%)	110 (53.9%)	0.550
Body temperature, median (IQR), °C	36.3 (36.0, 36.5)	36.2 (36.0, 36.5)	0.065
Oxyhemoglobin saturation, median (IQR), %	97.0 (96.0, 98.0)	97.0 (96.0, 98.0)	0.971
Days in FangCang hospital before enrollment, median (IQR), d	4.0 (3.0, 4.0)	4.0 (3.0, 4.0)	0.085
Patients source^*^			0.093
Centralized isolation site, *n* (%)	11 (11.3%)	20 (19.2%)	
Others, *n* (%)	193 (94.6%)	184 (90.2%)	
Place of birth			0.523
Wuhan, *n* (%)	170 (83.3%)	162 (79.4%)	
Hubei Province excluding Wuhan, *n* (%)	25 (12.3%)	33 (16.2%)	
Outside Hubei Province, *n* (%)	9 (4.4%)	9 (4.4%)	
Composite symptoms			
Fever, cough and fatigue, *n* (%)	112 (54.9%)	98 (48.0%)	0.166

*CM, Chinese medicine; IQR, interquartile range*.

* *Some patients were unknown source, but were not from the centralized isolation site, so they were categorized as “others.” There were 107 such patients in CM group cabin C and 100 in control group cabin B*.

Table 3 shows efficacy in each group based on primary and secondary outcomes. A significant difference in worsening rate was observed between groups (5/204 vs. 16/204, *P* = 0.014), RR = 0.312 (0.117, 0.837). No death occurred in either group. We performed a multivariate logistic regression analysis for the six risk factors: sex, age, group, patients source, place of birth and composite symptoms. The stepwise selection showed that after adjusting for the above factors, age (OR: 1.05; 95% CI: 1.00–1.09; *P* = 0.035) and patients source (OR: 3.42; 95% CI: 0.88–10.95; *P* = 0.050) were independent risk factors for worsening during treatment, and OR value of the group (treatment) (OR: 0.28; 95% CI: 0.09–0.76; *P* = 0.018) was <1, *P* < 0.05. The adjusted results (Table 4) showed that multiple logistic regression were consistent with the χ^2^ test results of previous primary outcomes in different treatment methods, and the worsening rate of the CM group was significantly lower than that of the control group.

**Table 3:** Primary and secondary outcome measurements during the trial.

	**CM group cabin C (*n* = 204)**	**Control group cabin B (*n* = 204)**	***P-*value**
**Primary outcome**			
Rate of worsening, *n* (%)	5 (2.5%)	16 (7.8%)	0.014
**Secondary outcome**			
Discharge			
Hospital stay time^*^, median (IQR), d	19.00 (15.8, 21.0)	18.5 (14.0, 21.0)	0.258
Discharge rate before clinical endpoint, *n* (%)	148(72.5%)	134(65.7%)	0.058
Composite symptoms remission			
Fever, cough and fatigue, *n*/*N* (%)	109/112 (97.3%)	96/98 (98.0%)	>0.999
Drug combination			
Patients taking Arbidol hydrochloride during the trial, *n* (%)	99 (48.5%)	110 (53.9%)	0.276
Patients taking CM after 7-day treatment, *n* (%)	104 (51.0%)	104 (51.0%)	>0.999

*CM, Chinese medicine; IQR, interquartile range*.

* *Hospital stay time: Cured patients*.

**Table 4:** Multivariate logistic regression analysis for the rate of worsening.

**Factors**	**Estimate**	**Std. error**	**OR (95% CI)**	***P*-value**
Group: treatment	−1.2615	0.5347	0.28 (0.09, 0.76)	0.018^*^
Age	0.0448	0.0212	1.05 (1.00, 1.09)	0.035^*^
Patients source: Centralized isolation site	1.2290	0.6279	3.42 (0.88, 10.95)	0.050

* *P < 0.05*.

**Figure 2:**
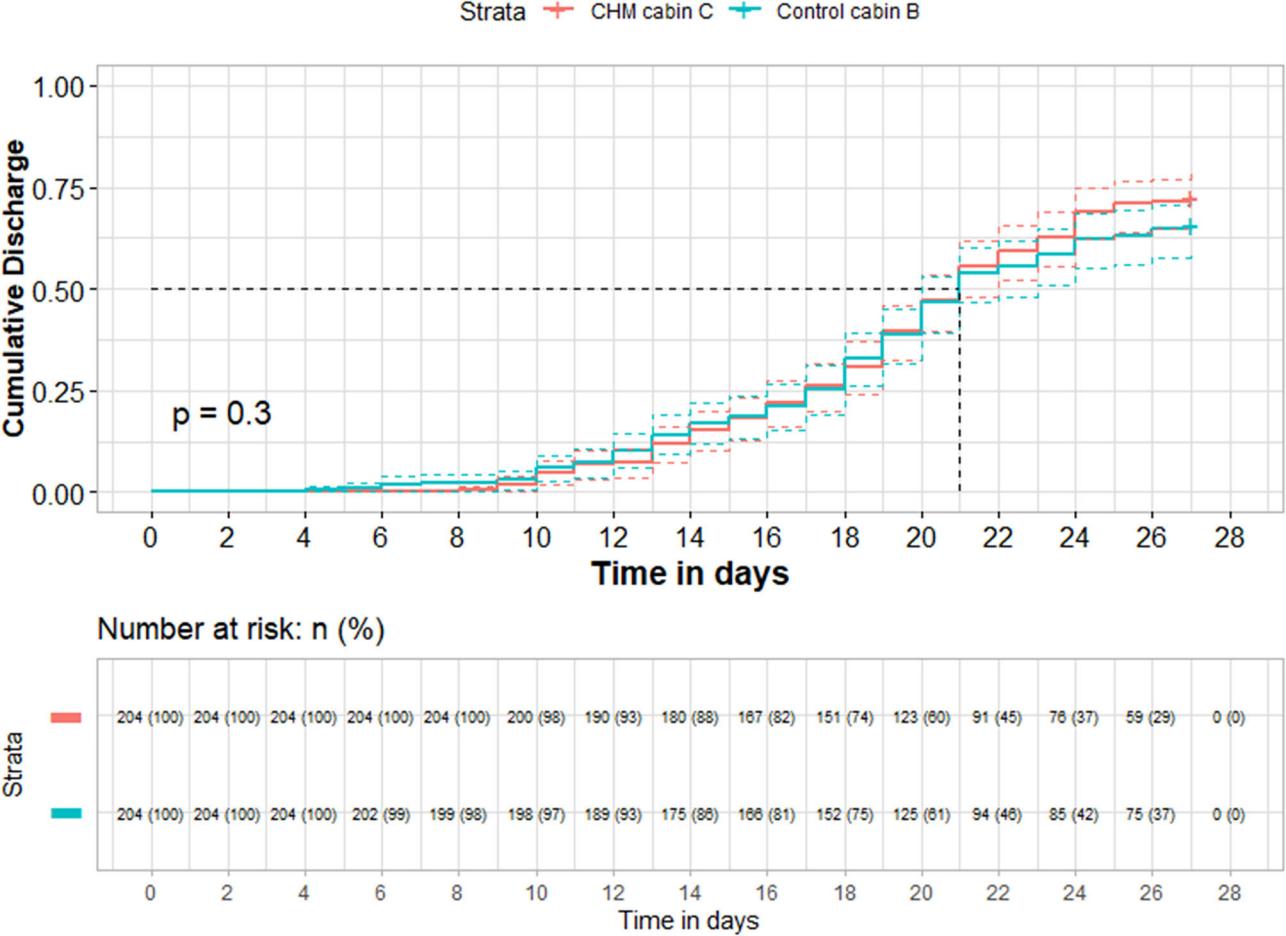
Cumulative incidence curves of discharge. +: Contained right censoring participants caused by death, worsening and transferring due to gradual closure of FangCang hospital.

148/204 (72.5%) participants in the CM group (cabin C) and 134/204 (65.7%) in the control group (cabin B) were cured and discharged before their clinical endpoint (*P* = 0.058). No significant difference was found in the median hospital stay time between groups (*P* = 0.258). Figure 2 shows hospital stay time survival curve of all discharged patients (*P* = 0.3). 51/204 participants in the CM group and 54/204 in the control group were transferred to other permanent hospitals due to gradual closure of the FangCang hospital. In almost all enrolled patients with any composite symptoms, these were improved after treatment, with no significant difference between groups (*P* > 0.999). No significant difference was found between the two groups in the number of patients taking Arbidol hydrochloride during the trial (*P* = 0.276), or in the number of participants taking CM in the follow-up period (*P* > 0.999).

Table 5 shows the incidence of adverse events in this trial. Eight cases of mild diarrhea with spontaneous remission occurred in total with no significant difference between groups (*P* = 0.151).

**Table 5:** CM granule-related adverse events.

	**CM group cabin C**		**Control group cabin B**		
**Adverse events**	**Participants, *n* (%)**	**Events, *n***	**Participants, *n* (%)**	**Events, *n***	***P-*value**
Diarrhea^a^	6/168(3.6%)	6	2/195(1.0%)	2	0.151

a *Patients without diarrhea at the time of enrollment were analyzed*.

## Discussion

This trial to investigate the effect of Huashibaidu granule on patients with mild, early stage COVID-19 was conducted in a FangCang hospital, dedicated to medical treatment of this disease. The results showed that adding Huashibaidu granule to early treatment could significantly reduce worsening in these patients. Several network pharmacology analysis studies had preliminarily explained the possible therapeutic mechanism of Huanshibaidu granule. A study found that Huashibaidu granule might treat severe COVID-19 through 45 potential target genes including 13 hub target genes (12). Other studies had found that quercetin and ursolic acid contained in Huashibaidu granule could inhibit COVID-19 by down-regulating IL-6 (13), quercetin and gallic acid could inhibit COVID-19 by down-regulating angiotensin converting enzyme II (ACE2) (14), and baicalein and quercetin were the top two compounds that had effect to treat COVID-19 mainly through TNF, PI3K–Akt and MAPK signaling pathway (15). However, compared with those who only received conventional treatment, the use of Huashibaidu granule did not significantly improve the short-term symptoms or affect patient discharge.

Previous studies had shown that COVID-19 can damage the organs of the nervous, circulatory, digestive and reproductive systems (16), and that prognosis of patients with end-stage COVID-19 was poor. Therefore, the issue of disease progression in patients with mild COVID-19 had attracted much attention. One study analyzed data from 61 COVID-19 patients and found that the risk factors for progression from early stage COVID-19 were age ≥ 50 years and the neutrophil to lymphocyte ratio ≥ 3.13 (17).

One study found that transmission of COVID-19 occurred mainly via contact within households (18). The establishment of FangCang hospitals aimed to limit development of the COVID-19 epidemic, by separating patients from healthy people and giving active treatment to patients. In our study, the rate of worsening in the two groups was far lower than indicated by the (11), and symptoms were alleviated in most symptomatic patients after 7 days treatment, though no significant difference was found between groups. These results illustrate the effect of FangCang hospitals in providing early active treatment for large numbers of patients with mild disease. These findings agreed with those of previous studies, two of which suggested that treatment with lopinavir-ritonavir within 12 days or remdesivir within 10 days from the onset of symptoms could promote recovery in patients with COVID-19 (19, 20). Analysis in another study suggested that treatment within 10 days after the onset of symptoms in COVID-19 patients was more effective than later treatment (21). In addition, patients with severe COVID-19 have been found to have more comorbidities (22). As one study had shown, COVID-19 patients with chronic obstructive pulmonary disease (COPD) have significantly higher mortality and worsening than those without COPD (23). In the FangCang hospital, concurrent treatment for diseases other than COVID-19 might also limit COVID-19 progression.

CM has a rich history in the treatment of epidemic infectious diseases. Ancient texts record large caldrons being used to boil large amounts of CM in public places during an epidemic, or soluble herbal or mineral drugs being placed into a well for large numbers of people to take, to achieve population-wide treatment or prevention. Our study in the FangCang hospital randomly allocated interventions within cabins, imitating the CM anti-epidemic approach. The difference was that we used CM granules meeting GMP criteria. In addition, with the support of good management in the FangCang hospital, we completed drug distribution, treatment adherence maintained, clinical endpoints observed and data recorded. The results of this study verified that the combined use of Huashibaidu granule and conventional treatment was better than conventional treatment alone in early treatment, supporting early treatment with Huashibaidu granule at well-managed medical locations for patients with mild COVID-19.

Our study has several limitations. Firstly, participants were allowed to take other CMs. It was unrealistic to completely restrict the use of various CMs that might improve condition in a FangCang hospital. Their use was allowed after the 7-day treatment period, however, and no significant difference between groups was found during the follow up period, suggesting that any use of CMs during that period did not have a significant effect on outcomes. Secondly, contrary to our intention, as shown in registration information, blood tests were not implemented on time during the trial. In emergency treatment conditions, the blood test was ordered preferentially for patients with worsening disease or those newly admitted to the FangCang hospital. This resulted in a lack of data collection for this secondary outcome. Thirdly, the trial was of unblinded design with no placebo for comparison with the Huashibaidu granule. Huashibaidu granule is a newly generated CM formula, so it was not feasible to create a stable placebo with similar appearance, smell and taste in the time frame of the study. Under the design of random allocation on cabin level, we needed to avoid the risk from nocebo effect caused by a failure blinding. In addition, the trial was conducted in a well-managed FangCang hospital in Wuhan, China. Although the results of this trial and other studies had shown the safety of Huashibaidu granule (24), it is still necessary to use Huashibaidu granule cautiously in complex clinical intervention programs. It is not recommended to use Huashibaidu granule rashly without good medical management and the guidance of experienced TCM doctors.

Our research indicated that 7 days of early treatment with Huashibaidu granule reduced worsening of mild COVID-19 among patients in the FangCang hospital. It supports Huashibaidu granule as an active option for early treatment of mild COVID-19 in similar well-managed medical locations.

## Data Availability Statement

The raw data supporting the conclusions of this article will be made available by the authors, without undue reservation.

## Ethics Statement

The studies involving human participants were reviewed and approved by Ethics Review Committee of Institute of Basic Research in Clinical Medicine, China Academy of Chinese Medical Sciences. The patients/participants provided their informed consent to participate in this study.

## Author Contributions

LHua, ZL, CL, and CZ: concept and design. LLi, WL, JW (5th author), JG, JW (17th author), JZ, YB, SC, YaC, YiC, XC, GD, LHu, JJ, LLe, BLi, DL, HL, JL, WQ, QM, HS, JS, BW, GW, WW, YXia, XXie, CX, MX, BY, JY, LZ, ZZ, HZ, YXio, and BLiu: acquisition, analysis, or interpretation of data. CZ: drafting of the manuscript. CZ, LLi, WY, and XXia: critical revision of the manuscript for important intellectual content. WY, XXia, and QC: statistical analysis. LHua: obtained funding. RH, YD, NS, CL, and YT: administrative, technical, or material support. WL, JW (5th author), JG, CL, ZS, and RC: supervision. All authors contributed to the article and approved the submitted version.

## Funding

This study was supported by National Key R&D Program of China (No. 2020YFC0841500). The funder of the study had no role in the study design, data collection, statistical analysis, results interpretation, or reports writing.

## Conflict of Interest

The authors declare that the research was conducted in the absence of any commercial or financial relationships that could be construed as a potential conflict of interest.

## Publisher's Note

All claims expressed in this article are solely those of the authors and do not necessarily represent those of their affiliated organizations, or those of the publisher, the editors and the reviewers. Any product that may be evaluated in this article, or claim that may be made by its manufacturer, is not guaranteed or endorsed by the publisher.

## Supplementary Material

Supplementary Material 1 The pharmacological actions and main effective substances of each CM in Huashibaidu granule.

## Abbreviations

CCEBTCM: China Center for Evidence-Based Traditional Chinese Medicine
CM: Chinese Medicine
COPD: Chronic Obstructive Pulmonary Disease
COVID-19: Coronavirus Disease 2019
GMP: Good Manufacturing Practice.
