# Supplementary material for: Chinese Medicine Formula Huashibaidu Granule Early Treatment for Mild COVID-19 Patients: An Unblinded, Cluster-Randomized Clinical Trial
Source: Front Med (Lausanne). 2021 Sep 16;8:696976. doi: 10.3389/fmed.2021.696976 (PMC8481869; doi:10.3389/fmed.2021.696976)
Supplement: Supplementary Material 1 — The pharmacological actions and main effective substances of each CM in Huashibaidu granule. [file Table_1.DOCX]

**Supplementary Material 1:** The pharmacological action and main effective substances of each CM in Huashibaidu granule

| **Table S1 Composition of Huashibaidu Granule** | | | | | |
| --- | --- | --- | --- | --- | --- |
| **Chinese phonetic name of CM** | **Latin name of CM** | **Content of crude drugs（g）** | **Representative ingredients** | **pharmacologic action** | **mechanism** |
| **Mahuang** | **Herba Ephedrae** | **6** | **Polysaccharide** | **anti-inflammatory** | **inhibits inflammation expression by regulating Factor-β1/Smad2 signaling** |
|  |  |  | **flavone** | **Anti-Oxidant** | **potent DPPH reduc-**  **ing activity.** |
|  |  |  | **catechin** | **antiviral** | **inhibited the acidification of ELS;**  **the growth of virus was inhibited** |
| **Kuxingren** | **Armeniacae Semen Amarum** | **9** | **Amygdalin** | **anti-inflammatory** | **exerts anti-inflammatory by suppressing the mRNA expressions of COX-2 and iNOS.** |
| **Shigao** | **Gypsum Fibrosum** | **15** | **Ca^2+^** | **heat-clearing** | **Lower PGE2 levels in the hypothalamus** |
|  |  |  |  | **anti-inflammatory** | **inhibits inflammation expression by reduce the expression of IL-β,TNF-α and IL-6** |
| **Gancao;** | **Glycyrrhizae Radix et Rhizoma** | **3** | **Glycyrrhizin** | **anti-inflammatory; oxidative stress** | **Binding ACE2 to prevent the infection of SARS-CoV-2;**  **Down-regulating proinflammatory cytokines;**  **Inhibiting the accumulation of intracellular ROS;**  **Inducing endogenous interferon to combat the SARS-CoV-2.** |
|  |  |  | **glycyrrhizic acid** | **Antiviral Activity** | **inhibit Virus invasion and replication;**  **stimulate antiviral innate immune responses;** |
| **Huoxiang** | **Pogostemonis Herba** | **10** | **Patchouli alcohol** | **Antiviral Activity;**  **anti-inflammatory** | **inhibit influenza Virus replication in vitro;**  **regulate the levels of inflammatory cytokines, including TNF-α, IL-10 and IFN-γ** |
| **Houpu** | **Magnoliae Officinalis Cortex** | **10** | **Magnolol** | **anti-inflammatory** | **decreasing the infiltration of neutrophils ;**  **reducing the production of pro-inflammation factors (IL-1β, TNF-α and MMP-9)** |
| **Cangzhu** | **Atractylodis Rhizoma** | **15** | **polysaccharides** | **immune response** | **NF-κB and Jak-STAT signaling pathways** |
| **Caoguo** | **Tsaoko Fructus** | **10** | **Essential oil** | **antibacterial** | **strong antibacterial activity in vitro** |
| **Fabanxia** | **Pinelliae Rhizoma Praeparatum** | **9** | **alkaloids** | **anti-inflammatory** | **inhibit the release of NO,TNF－α;**  **inhibit the expression of IL－8 and ICAM－1** |
|  |  |  | **polysaccharides** |  | **reducing the release of pro-inflammation factors (TNF-α,IL-1β and IL-6);**  **reduce the expression of IL-8 and ICAM-1** |
| **Fuling** | **Poria** | **15** | **polysaccharides** | **against oxidative stress;**  **anti-inflammatory** | **Up-regulation on ERK/Nrf2/HO-1 signaling pathway** |
| **Shengdahuang** | **Rhei Radix et Rhizoma** | **5** | **Emodin** | **anti-inflammatory** | **inhibiting the ERK and Wnt/β-catenin pathway;**  **downregulating the expression of a series of inflammatory mediators** |
|  |  |  | **polysaccharide** |  | **targeted MR and down-regulation of Th1-polarized immune response** |
|  |  |  | **Rhein** | **antibacterial** | **With antifungal activity and antimicrobial properties** |
| **Shenghuangqi** | **Astragali radix** | **10** | **Baicalin** | **antiviral activities** | **interacting with two catalytic residues, to prevent the peptide substrate approaching the active site.** |
|  |  |  | **baicalein** |  |  |
|  |  |  | **polysaccharide** | **anti-inflammatory** | **stimulating macrophage;**  **decreased levels of NO and TNF-α.** |
| **Tinglizi** | **Descurainiae Semen Lepidii Semen** | **10** | **helveticoside** | **anticancer** | **It has cytotoxic activity to cancer cell lines** |
| **Chishao** | **Paeoniae Radix Rubra** | **10** | **Paeoniflorin** | **oxidative stress** | **reverse the overexpression of NADPH oxidase 4** |

**CM, Chinese medicine.**
